# Editorial Comment: Intravesical injections of platelet-rich plasma is effective and safe in treatment of interstitial cystitis refractory to conventional treatment-A prospective clinical trial

**DOI:** 10.1590/S1677-5538.IBJU.2021.02.04

**Published:** 2021-02-03

**Authors:** Cássio L. Z. Riccetto

**Affiliations:** 1 Universidade Estadual de Campinas Faculdade de Ciências Médicas Divisão de Urologia Feminina Campinas SP Brasil Divisão de Urologia Feminina - Faculdade de Ciências Médicas da Universidade Estadual de Campinas – UNICAMP, Campinas, SP, Brasil

Jia-Fong Jhang ^1^, Teng-Yi Lin ^2^, Hann-Chorng Kuo ^1^

Neurourol Urodyn. 2019 Feb;38(2):703-709.

## COMMENT

In this study the authors assessed the effects of four monthly intravesical injections of 10 mL of autologous platelet-rich plasma (PRP) in forty patients (37 women and 3 men, aged 55.5 ± 11.1 years) with interstitial cystits / bladder pain syndrome (IC / BPS) who had previously failed to conventional treatments, including lifestyle modifications, cystoscopic hydrodistention, non-steroid anti-inflammatory drugs, oral Pentosan Polysulfate, tricyclic antidepressants, intravesical instillation of hyaluronic acid, or botulinum toxin A injection. Diagnosis of IC / BPS was based on symptoms and cystoscopic findings. Patients with Hunner's injury were not included. The Global Response Assessment (primary end-point) was 70% and 67.5%, after the 4th and 3 months after the 4th PRP injection, respectively. The O'Leary-Sant symptom score and visual analog scale score significantly decreased, while urinary frequency and episodes of nocturia decreased after PRP injections.

PRP is considered a relatively cheap and easy to prepare bio-compound. Platelets play a fundamental role in tissue regeneration, actively participating in the processes of mitosis, chemotaxis, differentiation and growth of pluripotent mesenchymal cells, in addition to inducing extracellular matrix production. The proteins contained in the alpha granules of the platelets have a strong influence on the reparative phenomena of wounds. These proteins include: platelet-derived growth factor (PDGF); transforming growth factor beta (“transforming growth factor” or TGF-ß); the platelet factor 4 (FP4); interleukin 1 (IL-1); platelet derived angiogenic factor (PDAF); vascular endothelial growth factor (“vascular endothelial growth factor” or VEGF); epidermal growth factor (“epidermal growth factor” or EGF); platelet-derived endothelial growth factor (“platelet derived endothelial growth factor” or PDEGF); epithelial cell growth factor (“epithelial cell growth factor” or ECGF); insulin-like growth factor (IGF) among others (1, 2).

Recent evidence confirms the great potential of using PRP in Plastic, Vascular surgery, Orthopedics, Trauma, Ophthalmology, Dermatology, Gynecology and Sports Medicine (3, 4). In Female Urology, PRP was previously proposed as a polypropylene mesh coating. If we assume that one of the most relevant pathophysiological mechanisms of interstitial cystitis is the increase in urothelial permeability related to proteoglycan deficiency, the use of PRP has a consistent rationale and may be clinically useful, mainly if other researchers are able to reproduce such results.
